# Editorial: Network physiology, insights into the brain system: 2021

**DOI:** 10.3389/fnetp.2022.1018142

**Published:** 2022-10-11

**Authors:** Klaus Lehnertz

**Affiliations:** ^1^ Department of Epileptology, University of Bonn Medical Centre, Bonn, Germany; ^2^ Helmholtz Institute for Radiation and Nuclear Physics, University of Bonn, Bonn, Germany; ^3^ Interdisciplinary Center for Complex Systems, University of Bonn, Bonn, Germany

**Keywords:** brain network, brain dynamics, brain pathologies, biological rhythms, sleep, network models, neuron models, synchronization

Due to its intricate structure and its tremendous functionality, the human brain is one of the most complex and fascinating dynamical systems in nature. The highly interconnected networks in the brain, which are neither random nor entirely regular, span multiple spatial scales, from individual cells and synapses *via* cortical columns to (sub)cortical areas. These networks support a rich repertoire of behavioral and cognitive functions, and in the case of brain pathologies, normal and abnormal functions and/or structures can coexist. Over the last 2 decades, concepts and quantitative methods of network science have significantly contributed to reveal fundamental principles of brain structure and function. By the same token, conceptualizing the brain as a network has led to a paradigm shift in establishing neurological and psychiatric disorders as network disorders, possibly paving the way to a better understanding of brain disorders and to more efficient treatment options. Still, network studies of the human brain, alongside with its aberrances, remain at an early stage. Limitations that may result from an isolated view of the brain are currently being addressed within the field of network physiology, that uses concepts and quantitative methods of network science to improve our understanding how organ systems dynamically interact. The goal of this special edition Research Topic of *Frontiers in Network Physiology–Networks in the Brain System* section, is to shed light on the progress made in the past decade in the *Networks in the Brain System* field and on its future challenges. This article collection–consisting of five original research articles, one hypothesis and theory article, and one perspective article–features contributions from leading experts that describe the state of the art, outlining recent developments and major accomplishments that have been achieved and that need to occur to move the field forward. The contribution by Czarnecki et al.. provides insight into how new neurons are recruited into memory traces during sleep. By means of *in silico* modeling of neuronal networks with scale-free coupling topology the authors identified a dichotomous network reorganization–mediated by the neuromodulator acetylcholine (ACh)—that can facilitate different aspects of memory formation and consolidation. The authors demonstrate a striking, state-dependent change in information flow throughout the network, with highly active hub cells integrating information in a high-ACh state, and transferring it to rest of the network in a low-ACh state. This result is corroborated by frequency-dependent frequency changes observed *in vivo* experiments.

Lehnertz et al. demonstrate the influence of ultradian and circadian rhythms on temporal changes of various characteristics of human brain dynamics: higher-order statistical moments and interaction properties of multiday, multichannel EEG signals as well as local and global characteristics of EEG-derived evolving functional brain networks. Their findings emphasize the need to take into account the impact of various biological rhythms in order to avoid erroneous statements about brain dynamics and about evolving functional brain networks.

Sawicki et al. report on the influence of music in a simulated network of FitzHugh-Nagumo oscillators with empirical structural connectivity obtained from healthy human subjects. The authors observe a frequency-dependent increase of coherence between the global network dynamics and the input signal induced by a specific music song, which compares to experimental findings of a global neural synchronization between different brain regions in the gamma-band and its increase immediately before transitions between different parts of the musical form. These findings may help to unravel the general modalities of the influence of music on the human brain.

Tripathi and Gluckman develop coarse-grained models of neural cell group activity (in the form of mechanistic neural mass models) that can reflect environmental input and experimentally measurable parameters, and reproduce normal and pathological dynamics. The developed neural mass models are parametrized through simple mathematical functions, show physiologically interpretable behaviors and dynamical transitions from one state to another, as a function of key parameters of the neural environment. The paper demonstrates the method and reveals key dynamics in small networks that are basic elements of epilepsy.

Tufa et al. report on a possible non-invasive biomarker of risk of sudden unexpected death in epilepsy (SUDEP), which is the leading seizure-related cause of death in people with epilepsy. The authors explored topological properties of cross-frequency functional brain networks derived from electroencephalograms recorded prior to, during and after seizures from people with epilepsy subdivided into SUDEP and non-SUDEP cohorts. Findings suggest a higher connectivity and more efficient flow of information in seizure networks from the SUDEP cohort.

The Hypothesis and Theory article by Goodfellow et al. discusses advancements in physical and mathematical modeling as well as in data analysis techniques that have potential to further advance our understanding of brain structure and function. The authors argue that progress in these fields could benefit from closer cross-disciplinary links in the cycle of model refinement and checking. They then offer two other concrete examples of neuroscience-inspired research that could make a reciprocal contribution back to neuroscience, namely research into photonic neurons as well as into chimera states.

The Perspective article by Sinha et al. centers around epilepsy as a network disease. Epilepsy is the most common chronic brain disease affecting more than 50 million people worldwide. It is characterized by seizures (either focal or generalized) that arise from aberrant activity in a distributed, large-scale network. The authors summarize the current state of knowledge and address several important challenges that could help to improve our understanding of the human brain in epilepsy.

I am confident that this article collection will inspire, inform and provide direction and guidance to researchers in the field.

